# ADP-ribosylation factor as a novel target for corneal neovascularization regression

**Published:** 2012-12-12

**Authors:** Chunyan Dai, Gaoqin Liu, Longbiao Li, Yanhui Xiao, Xueguang Zhang, Peirong Lu

**Affiliations:** 1 Department of Ophthalmology, the First Affiliated Hospital of Soochow University, China PR; 2 Jiangsu Institute of Clinic Immunology, the First Affiliated Hospital of Soochow University, China PR

## Abstract

**Purpose:**

To evaluate the roles of ADP-ribosylation factor (ARF) in alkali-induced corneal neovascularization (CNV).

**Methods:**

CNV was induced by alkali injury and compared in ARF1 inhibitor– or vehicle-treated mice 3 weeks after injury. Angiogenic and apoptosis factor expression in corneas after injury was quantified with reverse-transcription PCR. Human retinal endothelial cell apoptosis induced by ARF1 inhibitor was detected with flow cytometry.

**Results:**

The mRNA expression of ARF1 was augmented in the corneas after alkali injury. Compared with vehicle-treated mice, ARF1 inhibitor–treated mice exhibited impaired CNV 3 weeks after injury, as evidenced by corneal whole mount CD31-staining. Concomitantly, the enhancement of intraocular vascular endothelial growth factor expression was reduced in ARF1 inhibitor–treated mice compared to control mice after injury. Moreover, local administration of the ARF1 inhibitor after alkali injury enhanced intraocular caspase-3 expression. ARF1 inhibitor treatment can significantly induce human retinal endothelial cell apoptosis.

**Conclusions:**

The ARF1 inhibitor can induce the regression of alkali-induced CNV through increased endothelial cell apoptosis and downregulated intracorneal VEGF expression. ARF1 is an effective intervention target for CNV.

## Introduction

The cornea is characterized by the absence of blood vessels under physiologic conditions [1]. Corneal avascularity is required for optical clarity and optimal vision. Corneal neovascularization (CNV) arises from many causes, including corneal infections, misuse of contact lens, chemical burn, and inflammation, and can lead to severely impaired vision. CNV is also a high risk factor for graft rejection after allograft corneal transplantation [2]. Thus, developing effective measures for treating and preventing CNV is necessary.

ADP-ribosylation factors (ARFs) belong to the superfamily of Ras-related small GTPases and are believed to participate in vesicular transport and signal transduction events in the cell [3]. Six ARF members (ARF1–6) have been identified in mammalian cells, but ARF2 is not expressed in humans [4]. Similar to other Ras-related GTPases, the function of ARFs is regulated by their recycling between active GTP-bound and inactive GDP-bound conformations. ARF is activated by various growth factors, such as hepatocyte growth factor [5], colony stimulating factor-1 [6], and epidermal growth factor (EGF) [7]. Recent studies have demonstrated that the ARF family is a key regulator of tumor cell proliferation, migration, and invasion [7,8]. Endothelial cell migration and proliferation are important steps in the formation of CNV. Jones et al. [9] found that inhibiting ARF activity could reduce laser-induced choroidal neovascularization. However, ARF’s role in CNV has not been reported. In this study, alkali injury–induced experimental CNV was used to explore the effect of the ARF1 inhibitor on CNV and the mechanism of action .

## Methods

### Reagents and antibodies

The ARF1 inhibitor (SC-3030) was purchased from Santa Cruz Biotechnology (Santa Cruz, CA). Rat anti-mouse CD31 (MEC13.3) mAbs was purchased from BD PharMingen (San Diego, CA). Alexa Fluor 488 donkey anti-rat immunoglobulin G (Ig G) and the Alexa Fluor 488 annexin V/Dead Cell Apoptosis Kit (catalog no. V13241) were provided by Invitrogen Life Technologies (Carlsbad, CA). Sodium hyaluronate (HA) and Avertin (tribromoethanol) were purchased from Sigma-Aldrich Chemical Co. (St. Louis, MO). Human retinal endothelial cells (HRECs) were purchased from Y-J Biologic (Shanghai, China).

### Mice

Specific pathogen-free 6- to 7-week-old male BALB/c mice weighing 20 to 25 g were obtained from Shanghai SLAC Laboratory Animal Co. Ltd. (Shanghai, China) and were kept in our animal facility under specific pathogen-free conditions. All animal experiments were conducted in accordance with the Guideline for the Care and Use of Laboratory Animals of the Chinese Medical Academy and the Soochow University Animal Care Committee, and with the Association for Research in Vision and Ophthalmology Statement for the Use of Animals in Ophthalmic and Vision Research. Animals were kept in groups of five and fed regular laboratory chow and water ad libitum. A 12 h:12 h light-dark cycle was maintained.

### Alkali-induced corneal injury model

Mice were anesthetized at about 7 to 8 weeks old with an intraperitoneal injection of 1.8% (vol/vol) Avertin at a dose of 0.15 ml/10 g bodyweight. Corneal injury was induced by placing a 2 mm^2^ filter disc saturated with 1N NaOH onto the left eye of the mouse for 40 s, as previously described [10-14]. Alkali-injured mice were divided randomly into two groups. In the neovascularization enumeration or gene detection experiments, the alkali-treated eyes received 5 μl of ARF1 inhibitor dissolved in 0.2% sodium HA at a concentration of 25 μg/ml, or 5 μl of 0.2% sodium HA as vehicle twice a day for 2 weeks from day 7 after the alkali injury. At the indicated time intervals, the mice were killed. The corneas were removed and fixed in acetone for 20 min and then used for whole mount CD31 staining. In other experiments, the corneas were removed and placed immediately in RNAlater (Qiagen, Hilden, Germany), and kept at −86 °C until total RNA extraction was performed. Each experiment was repeated at least three times.

### Biomicroscopic examination

Eyes were examined with a slit lamp from Haag-Streit (BQ 900, Bern, Switzerland) and the results were photographed 3 weeks after alkali injury. Briefly, under anesthesia, photographs of the corneas were obtained using a digital camera (Nikon, Tokyo, Japan) linked to the slit lamp.

### Enumeration of corneal neovascularization

Corneal whole mount staining with CD31 was performed, and blood vessels in the corneas were measured according to previous reports [15]. The mice were killed using cervical dislocation, and the corneas were rapidly removed from the alkali-treated eyes; three to four relaxing radial incisions were made in the corneas. The corneal flat mounts were rinsed in phosphate buffered saline (PBS; 8 g NaCl, 0.2 g KCl, 1.44 g Na_2_HPO_4_, 0.24 g KH_2_PO_4_, pH 7.4), fixed in acetone, rinsed in PBS, soaked in 0.3% hydrogen peroxide solution to eliminate endogenous peroxidase, blocked in 2% donkey serum albumin, stained with rat anti-mouse CD31 (1:150; BD PharMingen) at 4 °C overnight, and washed in PBS. The corneas were then incubated with Alexa Fluor 488 donkey anti-rat immunoglobulin G (1/100) for 1 h at room temperature in the dark. Digital pictures of the flat mounts were taken, and the area covered by CD31 was measured morphometrically using NIH Image software (National Institutes of Health, Bethesda, MD). The total neovascularization area was then normalized to the total corneal area, and the percentage of the cornea covered by vessels was calculated. The relative neovascular area was compared between the treated group and the control group.

### Flow cytometric analysis of apoptosis

HRECs were cultured in Dulbecco’s modiﬁed Eagle’s medium supplemented with 10% fetal calf serum (FCS) in six-well plates at 1×10^5^ cells/well for 2 to 3 days in a humidified incubator at 37 °C in 5% CO_2_. After 24 h serum starvation, HRECs were cultured for 24 h in Dulbecco’s modiﬁed Eagle’s medium containing 10% FCS alone or with ARF1 inhibitor at a concentration of 14 μM, as previously reported [16]. Thereafter, cells were harvested and stained with the Dead Cell Apoptosis Kit with Annexin V Alexa Fluor 488 and propidium iodide dye; the cell apoptosis ratio was measured using a flow cytometer.

### Semiquantitative reverse-transcription polymerase chain reaction

Total RNAs were extracted from the corneas with the RNeasy Mini Kit (Qiagen, Tokyo, Japan) according to the manufacturer’s instructions. The resultant RNA preparations were further treated with RNase-Free DNase (DNase) I (Life Technologies Inc., Gaithersburg, MD) to remove genomic DNA. Two micrograms of total RNA were reverse-transcribed at 42 °C for 1 h in 20 μl of reaction mixture containing mouse Moloney leukemia virus reverse transcriptase and hexanucleotide random primers (Qiagen). Serially twofold-diluted cDNA was amplified for glyceraldehyde 3-phosphate dehydrogenase (GAPDH) to estimate the amount of transcribed cDNA. Then, equal amounts of cDNA products were amplified for the target genes using the primers under the following conditions: denaturation at 94 °C for 2 min, followed by the optimal cycles of 30 s at 94 °C, 30 s at 56–58 °C, 35 s at 72 °C, and a final 8 min extension step at 72 °C. The primers and PCR conditions used are shown in Table 1. Ten microliters of the amplified PCR products were fractionated on a 2.0% agarose gel and visualized with ethidium bromide staining. The band intensities were measured, and their ratios to GAPDH were determined with NIH Image Analysis software.

**Table 1:** Sequences of the primers used for reverse transcription polymerase chain reaction.

Gene name	Nucleotide sequence (5′→3′) sense/anti-sense	Annealing temperature (°C)	Product size (bp)	PCR cycles
*ARF1*	CGTGTGAACGAGGCCCGTGA	58	190	35
	TGGTGGCACAGGTGGCCT GA			
*Caspase-3*	ACCGGTGGAGGCTGACTTCCT	58	122	35
	GCGCGTACAGCTTCAGCATGC			
*VEGF*	GAGCGGAGCCGCGAGAAGTG	58	131	35
	TCCATGAGCCCGGCTTCCGA			
*GAPDH*	ACCACAGTCCATGCCATCAC	56	452	25
	TCCACCACCCTGTTGCTGTA			
*hARF1*	ATCGTGACCACCATTCCCAC	58	408	35
	GAGCTGATTGGACAGCCAGT			

In the table, hARF1 indicates human ARF1. GAPDH indicates human/mouse GAPDH primer.

### Statistical analysis

The means and standard error of the mean (SEM) were calculated for all parameters determined in the study. Values were processed for statistical analyses (Student *t* test) with statistical software SPSS 15.0 (SPSS Inc., Chicago, IL) p<0.05 was considered statistically significant.

## Results

### Intracorneal ADP-ribosylation factor 1 mRNA expression after alkali injury

**Figure 1:**
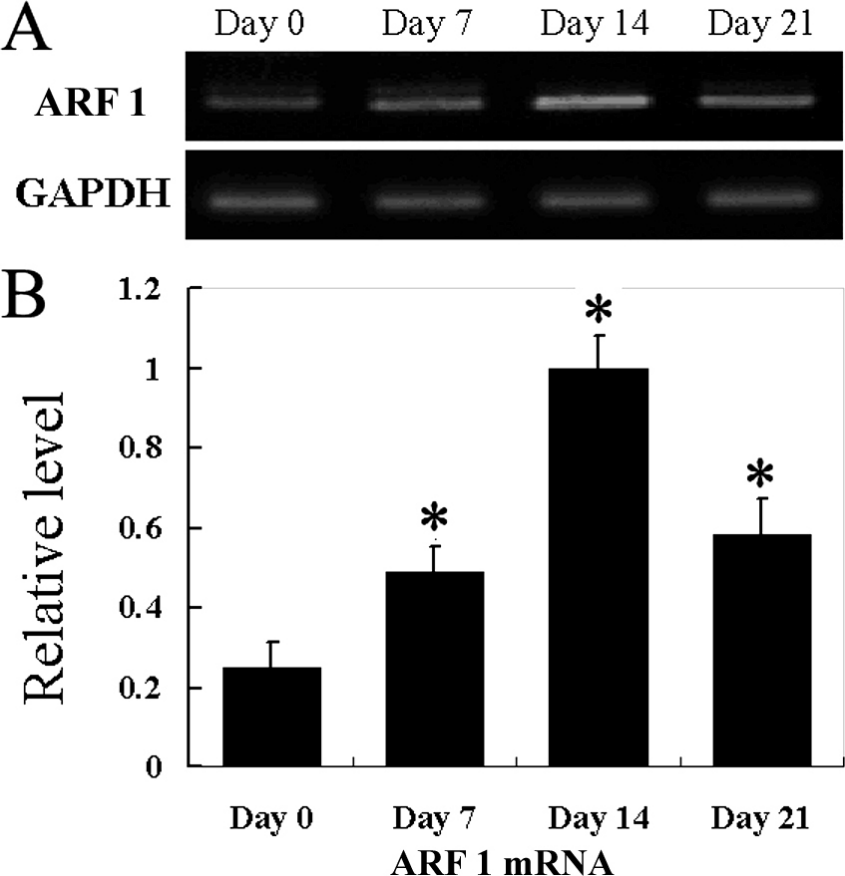
ADP-ribosylation factor 1 mRNA expression in corneas after alkali injury. **A**: Semiquantitative reverse transcription polymerase chain reaction (RT–PCR) was performed to evaluate mRNA (mRNA) expression of ARF1. Corneas were harvested at the indicated time points, and five corneas at each time point were pooled to extract total RNAs. Representative results from three independent experiments are shown. **B**: The relative level of ARF1 gene expression to glyceraldehyde 3-phosphate dehydrogenase (GAPDH) was determined. All values represent the mean±standard error of the mean (SEM) of three independent measurements (*p<0.05 versus day 0).

ARF1 mRNA expression in corneas after alkali injury was examined with reverse-transcription PCR (RT–PCR). ARF1 mRNA was faintly detected in the normal corneas, but was markedly increased in the injured corneas at day 7, 14, and 21 after alkali injury, and reached a peak on the 14th day (Figure 1A, B).

### Effect of ARF1 inhibitor on alkali-induced corneal neovascularization

**Figure 2:**
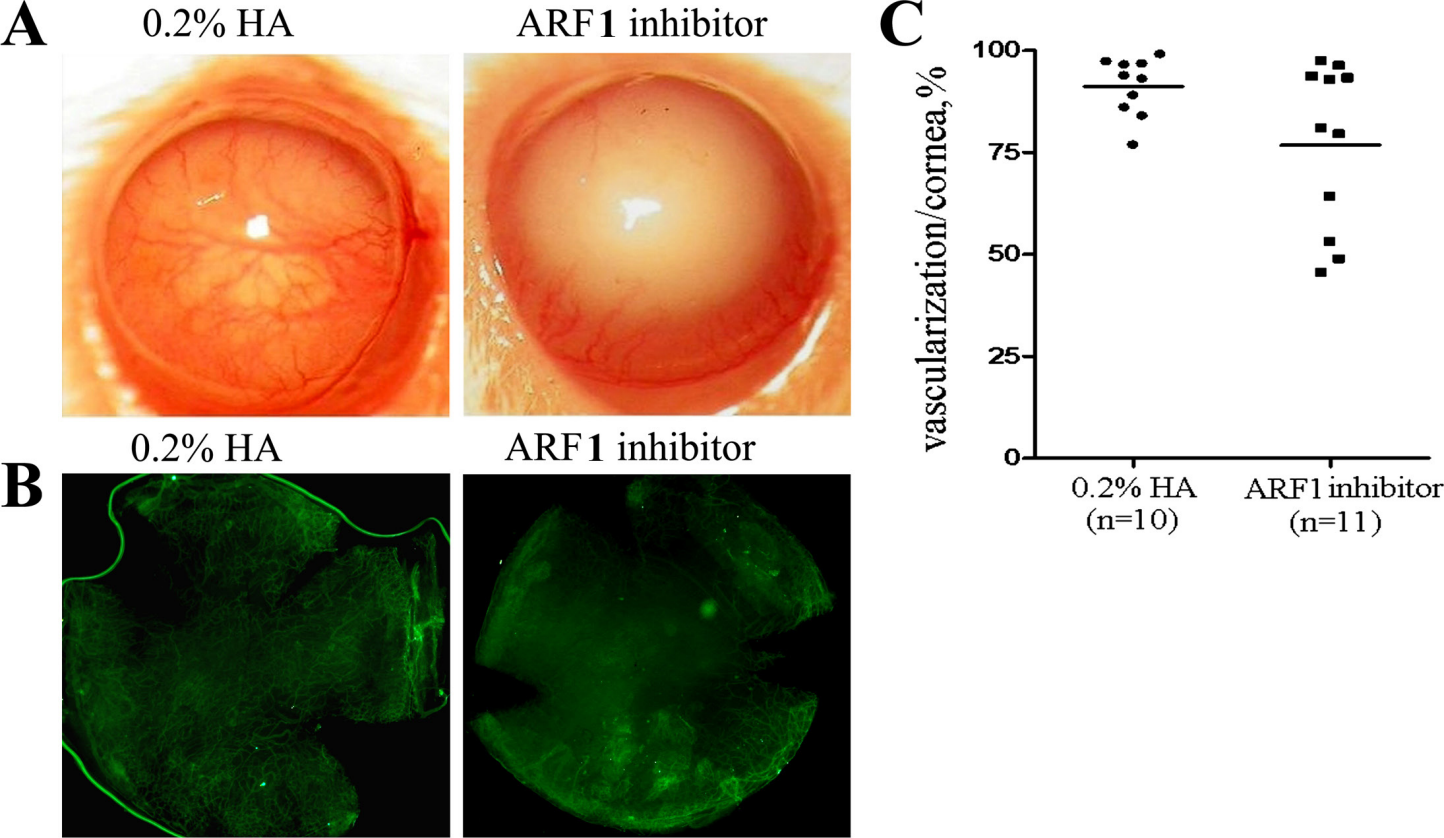
ARF1 inhibitor local administration on alkali-induced corneal neovascularization. One week after the alkali burn, the mice were divided randomly into two groups. The treated group received ARF1 inhibitor for 2 weeks, and the control group received 0.2% HA. **A**: Representative macroscopic observation of corneal neovascularization (CNV) 3 weeks after alkali injury are shown. **B**: Representative immunofluorescences of corneal microvessel densities in different treatment group 3 weeks after alkali injury are shown. **C**: The distribution of the percentage of CNV in each group are shown here (horizontal line represents the average; *p<0.05 versus the control group).

The effect of the ARF1 inhibitor on alkali-induced CNV was explored. In the alkali-induced CNV model, CNV was formed at 1 week after the alkali injury. From day 7 after alkali injury, the mice were locally administered 25 μg/ml of the ARF1 inhibitor in 0.2% HA or 0.2% HA as vehicle twice per day for 2 weeks. Microobservation concerning the CNV 3 weeks after alkali burn showed that CNV had significantly decreased in the ARF1 inhibitor–treated group compared to the control group (Figure 2A).

To further observe the effect of the ARF1 inhibitor on CNV, corneal whole mount staining by CD31—a neovascular marker—was performed. The ARF1 inhibitor–treated group showed a small number of thinner new blood vessels compared to the control group. The CNV areas in the treated group were significantly smaller than those of the control group (p<0.05), suggesting that the ARF1 inhibitor induced regression of newly formed vessels in the corneas (Figure 2B, C).

### Enhanced intracorneal caspase-3 and reduced vascular endothelial growth factor messenger ribonucleic acid expression after ARF1 inhibitor intervention

**Figure 3:**
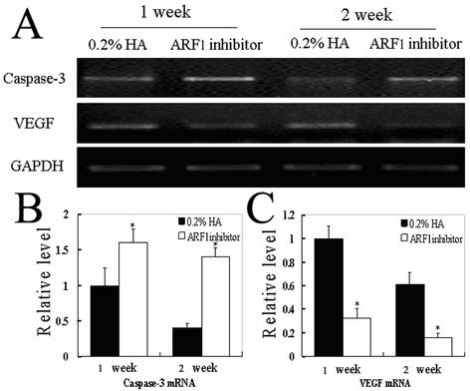
The caspase-3 and vascular endothelial growth factor (VEGF) gene expression in corneas after ARF1 inhibitor local administration. **A**: Representative reverse transcription polymerase chain reaction (RT–PCR) of caspase-3 and VEGF expression 1 and 2 weeks after ARF1 inhibitor-treated and control group are shown. **B** and **C**: The relative levels of caspase-3 and VEGF gene expression to GAPDH were determined. All values represent mean±standard error of the mean (SEM) of three independent measurements (*p<0.05 versus 0.2% HA).

Vascular endothelial cell proliferation or apoptosis is a critical step in angiogenesis. Vascular endothelial growth factor (VEGF) is a major mediator of CNV, and caspase-3 is the main factor in apoptosis. Caspase-3 and VEGF expression was detected with RT–PCR at 1 and 2 weeks after ARF1 inhibitor intervention. The results showed that the ARF1 inhibitor–treated group exhibited significantly increased intracorneal caspase-3 mRNA expression and decreased intracorneal VEGF mRNA expression compared with the control group (p<0.05), suggesting that the ARF1 inhibitor upregulated caspase-3 expression and downregulated VEGF expression in the corneas after alkali burn (Figure 3A-C).

### ADP-ribosylation factor 1 inhibitor induced apoptosis in human retinal endothelial cells

**Figure 4:**
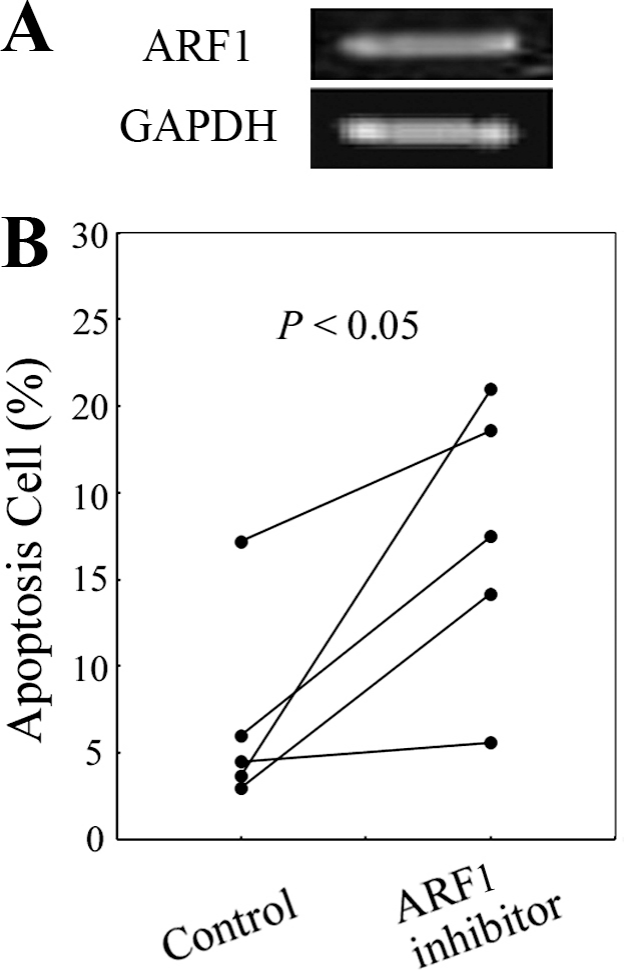
Enhanced human retinal endothelial cell apoptosis after ARF1 inhibitor treatment. **A**: Representative reverse transcription polymerase chain reaction (RT–PCR) of ARF1 expression on cultured human retinal endothelial cells (HRECs) is shown here. **B**: HRECs were cultured in six-well plates in Dulbecco’s modiﬁed Eagle’s medium containing 10% fetal calf serum (FCS) alone or ARF1 inhibitor (14 μM) for 24 h. The cell apoptosis rates in each sample were determined as described in the Methods section. The results from five independent experiments are shown. Each symbol represents the percent of cell apoptosis in each group. The Student *t* test (two-tailed) was used.

To determine whether the effect of the ARF1 inhibitor on CNV is caused by endothelial cell apoptosis, ARF1 expression was detected with RT–PCR performed using the total RNA from the cultured HRECs (Figure 4A). The effect of the ARF1 inhibitor on the HRECs was further determined with flow cytometry. HRECs were exposed to Dulbecco’s modiﬁed Eagle’s medium containing 10% FCS alone in the presence or absence of 14 μM of the ARF1 inhibitor. After 24 h, the ratio of apoptosis cells (Figure 4B) in the ARF1 inhibitor–treated group (17.34±1.61%) was significantly increased compared to the control group (6.88±1.18%; p<0.05), suggesting that the ARF1 inhibitor promoted vascular endothelial cell apoptosis and then induced CNV regression.

## Discussion

ARF, a subfamily of small GTP-binding proteins in the Ras superfamily, was initially named for its ability to function as a cofactor in the ADP-ribosylation of Gs by cholera toxin [17]. ARF protein is widely present in eukaryotic cells such as yeast, *Drosophila*, Arabidopsis, and humans. ARF is an important regulator of vesicle transport factors, and acts as a “molecular switch” in cellular signal transduction. However, whether ARF is involved in CNV is still unclear. In the current study, intracorneal ARF1 expression was first detected after alkali injury using RT–PCR. The ARF1 mRNA was significantly increased, peaking on the 14th day after the alkali burn. This result prompted further investigation of the role of ARF1 in developing experimental CNV.

CNV development is a dynamic and complex process. Tissue injury induces the expression of various growth factors, cytokines, and chemokines. They regulate vascular endothelial cells (VECs) proliferation, survival, migration, differentiation, and lumen formation. VEC proliferation and apoptosis play important roles in vascular formation and degradation, and we previously observed that CNV was formed at 1 week after alkali injury [10]. In the current study, local administration of the ARF1 inhibitor was initiated 1 week after alkali injury.

Apoptosis is critical for various physiologic and pathological processes. Two principal pathways exist for initiating apoptosis. One is the death receptor pathway, also termed the “extrinsic” pathway. The other is the mitochondrial or “intrinsic” pathway. Despite their differing modes of initiation, both pathways have the same outcome: activating a cascade of proteolytic enzymes, members of the caspase family. Caspase-3 is a key molecule in the apoptotic process. Recent studies on the ARF protein function have mainly focused on tumor cells. The reported data suggest that ARF could contribute to tumor cell progression and invasion by controlling the phosphatidylinositol 3-kinase and mitogen-activated protein kinase pathways, respectively [7]. ARFs also regulate phospholipase D (PLD) [18,19], suggesting that PLD might mediate some effects of ARF. PLD activity is closely related to cell proliferation and apoptosis. Birbes et al. [20] observed that PLD had an antiapoptotic effect on ECV304 cells; however, inhibiting PLD activity contributed to cell apoptosis. The published data indicate that destroying the cytoskeletal structure could induce cell apoptosis [21]. ARFs regulate cell migration through actin cytoskeleton rearrangement. Inhibiting VEC apoptosis can promote angiogenesis, while inducing VEC apoptosis can inhibit angiogenesis and then lead to vascular regression. In our study of alkali injury–induced CNV, the RT–PCR results showed that expression of the apoptosis-related factor caspase-3 was significantly increased in the corneal tissue of the ARF1 inhibitor–treated group in relation to the control group. To further determine the mechanism of antiangiogenic activity in the ARF1 inhibitor, the effect of the ARF1 inhibitor on HRECs was observed. The results from flow cytometric analysis showed that ARF1 inhibitor treatment promoted cell apoptosis of HRECs. Thus, the ARF1 inhibitor induced VEC apoptosis to promote CNV regression.

VEGF is a critical mediator in endothelial survival, proliferation, migration, and formation of the vascular tube. Previous studies have shown that VEGF promotes ARF activation, thus controlling endothelial cell migration and angiogenesis [22,23]. Moreover, ARF expression is markedly upregulated in association with an increase in capillary density in a mouse hind limb ischemia model of angiogenesis [22]. In this study, the RT–PCR results showed that VEGF expression was decreased in the corneal tissue of the treated group compared with the control group. The ARF1 inhibitor inhibited neovascularization by downregulating VEGF expression.

In summary, the present study showed that ARF1 is closely related to the formation of CNV. ARF1 affected angiogenesis-related factor (such as VEGF) expression and regulated cell apoptosis to influence the formation of CNV. Although the exact mechanism of ARF1 in alkali burn–induced CNV requires further elucidation, the preliminary results reported here show that ARF1 can be used as an effective intervention target against ocular neovascularization.
